# What to Observe at the Bed-side and after Death in Medical Cases

**Published:** 1853-05

**Authors:** 


### REVIEWS.

---

Art. VIII.—*What to Observe at the Bedside and After Death in Medical Cases.* Published under the authority of the London Medical Society of Observation. Philadelphia, Blanchard & Lea, 1853, 8vo., pp. 202.

Here is offered to the profession a manual, the salutary influence of which will be felt in all time to come. One cannot help wondering, after such a work has been brought forth, why it was so long appearing. A guide so convenient and useful, so necessary, indeed, to uniformity of observation, we would think would have been long ago suggested to the mind of some ingenious author. We hail its appearance with sincere pleasure, and beg every reader who aspires to become accurate in observation, and especially every one who has any thought of ever communicating his observations to the world, to lose no time in obtaining a copy.

As a specimen of the manner in which it proposes to have diseases studied, we subjoin the following extract:

*Throat, etc. Uvula:*—Length; form; thickness; direction of its axis;—surface, smooth, glazed; color—œdematous; flaccid; abscess sloughing; ulcers; vesicles; exudation, etc.

*Soft Palate:*—(Particulars as *Uvula*).

*Fauces:*—Size of opening ; state of pillars (particulars as *Uvula*).—*Pain:* its direction ;—other sensations, constriction, dryness, etc.;—tactile sensibility, increased, diminished.—Noisy respiration; snoring.

*Tonsils:*—Swelling and tenderness externally —Position of tonsils; distance between them;—size; form; consistence; fluctuating;—tender to touch;—color;—surface; dry, moist, smooth, glazed, uneven; openings on surface;—œdema; abscess; sloughing; ulcers; secretions and exudations on surface.

*Pharynx:*—Size of cavity;—apparent thickness of membrane; smooth, glazed, granulated; dry, moist;—vessels apprently full;—its color;—"stains."—Secretions; exudations; blood on surface.—Œdema; bogginess; fluctuation; abscesses; tumors; sloughing; ulcers, and other destructions of substance; cicatrices.—Condition of follicles.—Projections from posterior nares into cavity.—*Abnormal sensations* in pharynx; burning, dryness, constriction, etc. Tenderness.

*Œsophagus:*—Swelling in neck externally.—Examination with bougie; obstruction to passage, its seat; can it be overcome by moderate pressure?—Signs of dilatation and sacculation?—Is any thing visible during retching?—*Pain* in course of gullet, during swallowing, or at other times;—sense of constriction, burning, globus, etc.

*Deglutition:*—Pain during the act: its seat, extent, duration, character.—Deglutition difficult; period of act at which difficulty is perceived;—attempt to swallow productive of sense of suffocation, or spasm of muscles of throat or pharynx;—is difficulty greatest with solids or liquids?—what matters are swallowed most easily?—is a large gulp of liquid swallowed more easily than a small one?—has the patient any contrivance for rendering the act easier?—does the act produce cough?—do swallowed matters return by nostrils?—is the act affected by temperature of matters?—is the site of obstruction distinct to patient, and how?—is deglutition favored by a recumbent posture? Frequently or constantly repeated efforts at deglutition.

*Regurgitation:*—Period after swallowing at which it occurs; attended with effort, anxiety, or nausea? accompanied by any stethoscopic sound?—Physical characters of regurgigated matters; their reäction.

